# Disseminated Coccidioidomycosis Following COVID-19 Mimicking Metastatic Thoracic Relapse of Well-Differentiated Liposarcoma: A Case Report

**DOI:** 10.3389/fmed.2021.715939

**Published:** 2021-09-13

**Authors:** Elise F. Nassif, Nolan Maloney, Anthony P. Conley, Emily Z. Keung

**Affiliations:** ^1^Department of Surgical Oncology, The University of Texas MD Anderson Cancer Center, Houston, TX, United States; ^2^Department of Pathology, The University of Texas MD Anderson Cancer Center, Houston, TX, United States; ^3^Department of Sarcoma Medical Oncology, The University of Texas MD Anderson Cancer Center, Houston, TX, United States

**Keywords:** COVID 19, coccidioidomycosis, lymphopenia, metastasis, sarcoma

## Abstract

**Introduction:** COVID-19 is associated with immune dysregulation which may increase susceptibility to atypical infectious diseases, particularly in the vulnerable cancer patient population. Coccidioidomycosis is an endemic fungal infection which presents with mild-to-moderate pneumonia in most cases.

**Case Presentation:** The presented case is a 67-year-old woman living in the southwestern United States who is under close observation for well-differentiated liposarcoma of the abdominal wall. She presented with persistent cough and fatigue following COVID-19 infection. Imaging revealed new pulmonary nodules, a chest wall mass and bone lesions. The imaging appearance of these lesions was consistent with metastatic disease, although distant metastasis is not typical in well-differentiated liposarcoma. Biopsy of the chest wall mass revealed granulomatous fungal infection and serology was positive for coccidioidomycosis. At the time of diagnosis, the patient was lymphopenic, possibly a sequela of recent COVID-19 infection and which may have contributed to the development of her atypical disseminated form of coccidioidomycosis. Patient was treated with fluconazole for the coccidioidomycosis and continued observation for mild progression of the liposarcoma. On follow-up imaging, the chest wall mass and lung nodules have decreased in size and the patient remains on antifungal treatment. There has been no further increase in the liposarcoma mass.

**Conclusion:** COVID-19 may be associated with increased risk of atypical forms of infectious diseases in cancer patients, which physicians should be aware of before giving systemic treatments for cancer. In endemic regions, co-infection by coccidioidomycosis should be suspected in cases of persistent symptoms after COVID-19 infection.

## Introduction

COVID-19 as well as cancer may cause immune dysregulations, placing patients at greater risk of atypical infectious diseases. COVID-19 induces lymphopenia, which may increase susceptibility to rare and more severe forms of systemic infections (1).

Coccidioidomycosis is a fungal infection, resulting in a mild-to-moderate pulmonary infection in most cases. It is an endemic infection in the southwest regions of the United States of America and some regions of Central and South America. The incidence rate of this infection has been increasing over the past decade. In very rare cases the disease can be disseminated and cause osteomyelitis, synovitis, lymphadenitis, soft tissue infections, cutaneous disease, peritonitis or meningitis (2).

COVID-19 and coccidioidomycosis have shared risk factors and overlapping symptoms, making the diagnosis of coccidioidomycosis more challenging and potentially resulting in delay in treatment of the fungal infection (3). Moreover, coccidioidomycosis may present atypically in the setting of COVID-19 induced lymphopenia.

We present the case of disseminated coccidioidomycosis with lung, soft tissue and bone lesions mimicking metastatic relapse of well-differentiated liposarcoma.

## Case Description

### Patient Information

**Figure 1:**
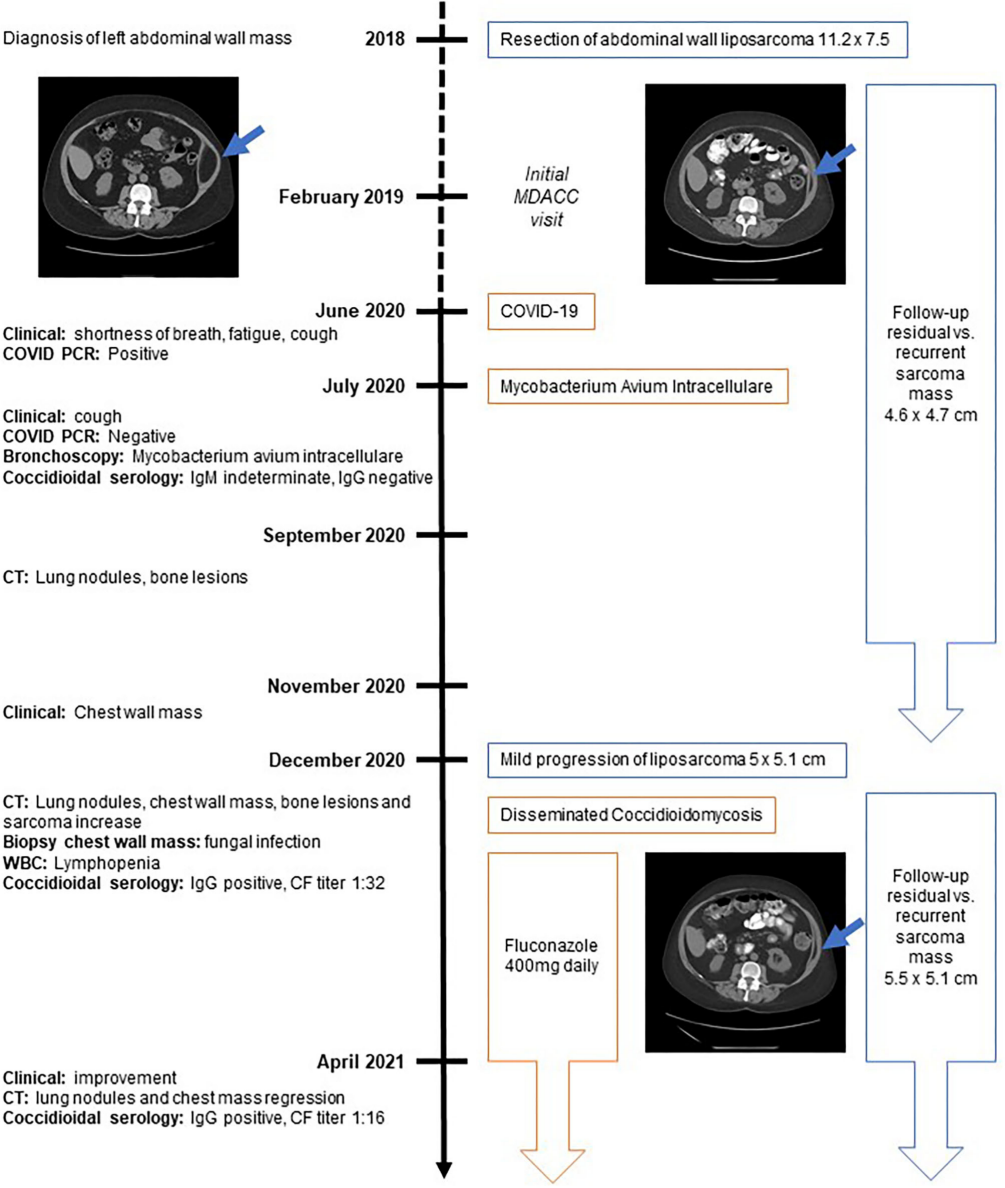
Timeline. Timeline displaying symptoms and diagnostic assessments on the left side of the figure. The right side displays diagnosis and therapeutic management of cancer (in blue) and infectious diseases (in orange). CF, Complement fixation; CT, computed tomography; PCR, polymerase chain reaction; WBC, white blood cell count.

The reported patient is a 67-year-old female who resides in the southwestern United States with notable past medical history of asthma and asbestosis. Notably, there was no history of immunosuppression nor infectious diseases prior to the present episode. She underwent resection of a 11.2 × 7.5 cm well-differentiated liposarcoma of the abdominal wall in July 2018. At the time of initial evaluation at our center in February 2019, she was noted to have a 4.6 × 4.7 cm recurrent vs. residual focus of disease between the left internal oblique and left transversus abdominus muscles for which the patient remains in observation (Figure 1).

### Informed Consent

The patient has given informed consent for publication of this case.

### Clinical Findings

In June 2020, the patient developed shortness of breath, increased fatigue and cough. No fever was documented or reported. An initial COVID-19 polymerase chain reaction test was obtained by the patient's local physician and was positive. No further laboratory or imaging evaluation was obtained at that time point. Multiple subsequent COVID-19 tests were negative, however, the patient's symptoms persisted.

A bronchoscopy was performed in July 2020 and the patient was diagnosed with an atypical mycobacterial infection (mycobacterium avium intracellulare) for which antibiotics were not recommended. At that time, the coccidioides serology was indeterminate for IgM, negative for IgG and antibody titers were negative.

In September 2020, symptoms persisted and computed tomography (CT) imaging revealed subcentimeter pulmonary nodules and small sclerotic bony lesions. As the lesions were too small to biopsy, close interval follow-up was recommended.

**Figure 2:**
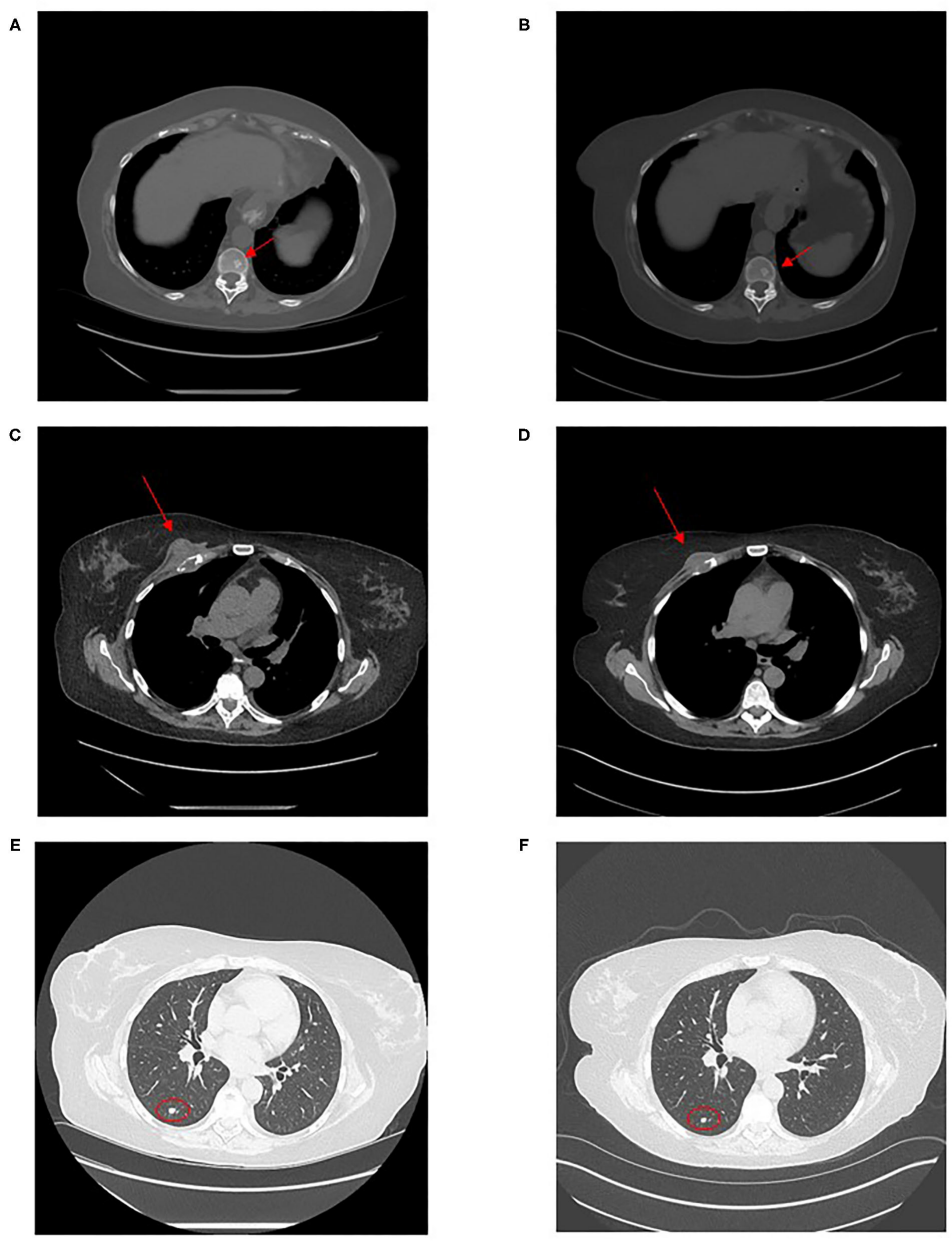
Computed tomography images of disseminated coccidioidomycosis lesions. Computed tomography images show: an axial sclerotic bone lesion in December 2020 **(A)** and after 4 months of fluconazole treatment in April 2021 **(B)**, the chest wall mass in December 2020 **(C)** and in April 2021 **(D)** and a right lower lobe lung nodule in December 2020 **(E)** and in April 2021 **(F)**.

In November 2020, the patient palpated a new right chest wall mass. She reported pulmonary symptomatic improvement of prior respiratory symptoms and was without fever or systemic inflammatory symptoms. Follow-up CT imaging demonstrated new bilateral pulmonary nodules, a 4.8 × 2.7 cm right anterior chest mass extending from the pectoralis major muscle into the costochondral junction of the right third rib, areas of sclerotic foci in axial bones and a slow progression of the known left abdominal wall recurrent vs. residual liposarcoma measuring 5.5 × 5.1 cm (Figures 2A,C,E).

### Diagnostic Assessment

The appearance of new lung nodules, bone lesions and chest wall mass in this patient with liposarcoma was suspicious for distant metastatic lesions. The patient thus presented to our institution in advance of her usual surveillance visit for further evaluation. Although the radiographic features of these lesions were consistent with metastatic disease, well-differentiated liposarcomas are only rarely known to distantly metastasize. A biopsy was therefore recommended to confirm the diagnosis of metastatic liposarcoma prior to initiation of chemotherapy.

**Figure 3:**
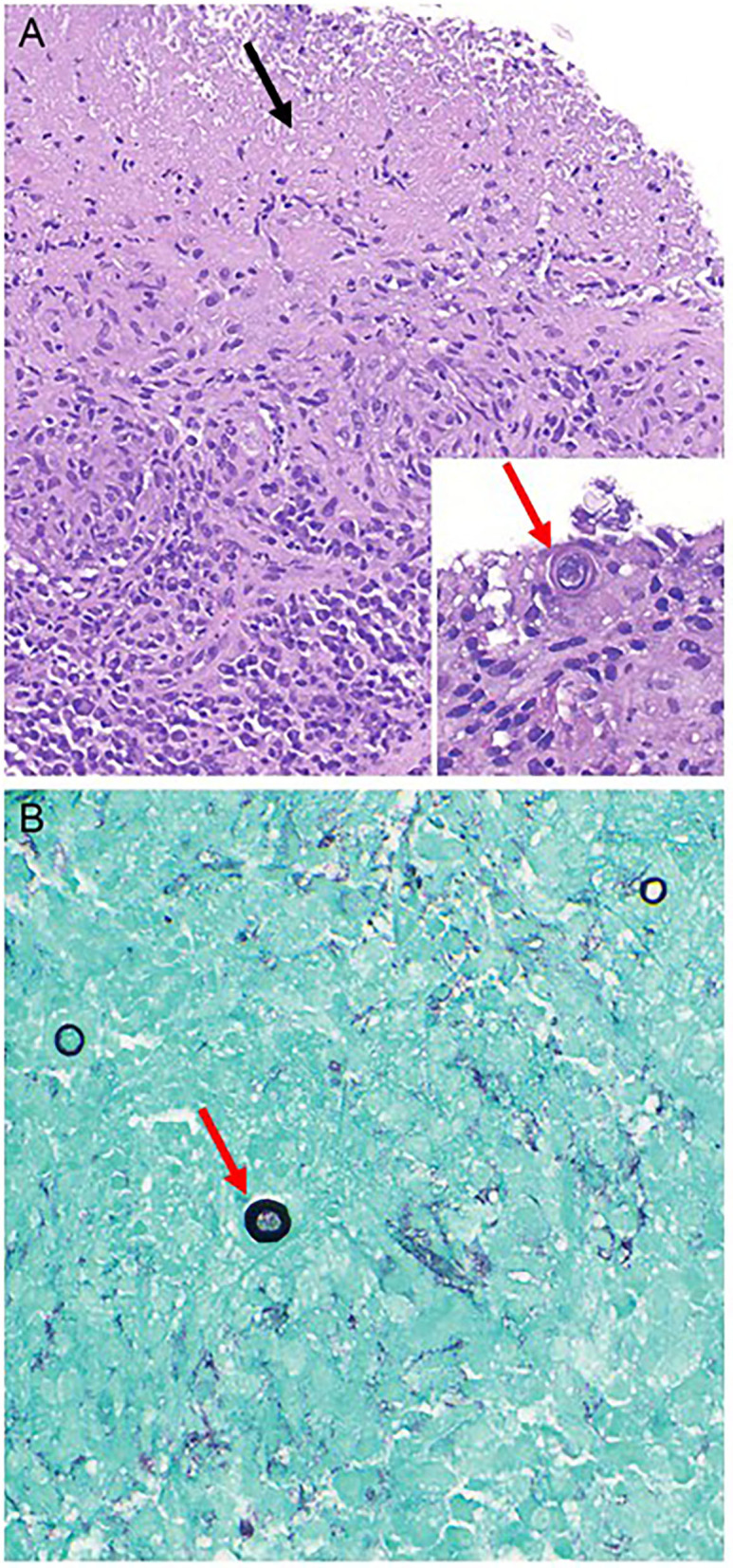
Histologic images of chest wall mass biopsy. Hematoxylin & Eosin stained microscopic sections show a diffuse granulomatous inflammation with areas of necrosis (**A**, 100x, black arrow pointing to necrosis). Rare intact organisms on are consistent with immature spherules of *Coccidioides immitis* (**A**, inset, 400x, red arrow pointing to organism). Grocott's methenamine silver stain highlights scattered spherical structures suggestive of yeast forms (**B**, 400x, red arrow pointing to organism).

In December 2020, a core needle biopsy of the right chest wall mass was performed. The patient developed a right chest wall cellulitis post-biopsy which was treated and resolved with Doxycycline. The pathological examination found no evidence of malignant cells but revealed granulomatous inflammation with necrosis and rare spherical structures highlighted by Grocott's Methenamine Silver stain suggestive of fungal organisms (Figures 3A,B).

White blood cell count displayed a lymphopenia with a lymphocyte count of 430/mm^3^. Serology and antigen for cryptococcus were negative, as was the intra-dermal tuberculosis test.

Serological tests for coccidioidomycosis were negative for IgM and positive for IgG. Coccidioidomycosis complement fixation (CF) titer was 1:32.

### Therapeutic Intervention

Systemic anti-fungal treatment with oral fluconazole 400 mg daily was initiated with close follow-up and imaging. Patient remains under treatment with good tolerance. No other therapeutic intervention has been required.

### Follow-Up and Outcomes

In April 2021, patient reported persistent asthenia and shortness of breath but no cough. CT imaging showed decrease in lung nodules and chest wall mass which now measured 3.2 × 1.3 cm (Figures 2D,F). The sclerotic bony lesions were unchanged (Figure 2B). The residual abdominal wall liposarcoma was stable in size comparison to December 2020, with slight increase compared to postoperative imaging in February 2019. CF titer was 1:16. Lymphocyte count was 740/mm^3^.

The patient continues fluconazole treatment for an additional 6 months before repeat imaging and clinical follow-up.

## Discussion and Conclusions

COVID-19 pandemic has affected cancer care mainly through diagnostic and therapeutic delays (4). COVID-19 may also contribute to further immune suppression in this patient population and predispose to atypical systemic infections (1). Misdiagnosis of atypical infectious diseases, particularly in the uniquely vulnerable oncology population, could potentially be fatal. Tissue biopsy was necessary to elucidate the etiology of new, progressive and multifocal lesions and importantly led to the avoidance of potentially myelosuppressive chemotherapy for presumed metastatic liposarcoma and to the initiation of appropriate antimicrobial treatment disseminated coccidioidomycosis.

In endemic regions, the rate of disseminated coccidioidal infection remains extremely rare, even among cancer patients (5). Moreover, in the present report, the patient did not receive systemic chemotherapy and the well-differentiated liposarcoma burden of disease was low volume and stable, making it unlikely that the disseminated coccidioidal infection would be triggered solely by the oncologic history. In this case, the patient's initial COVID-19 infection and induced lymphopenia may have contributed to the extent of the coccidioidal disease. However, COVID-19 and coccidioidomycosis have overlapping risk factors and symptoms and no definite causal relationship can be attributed to the sequence of events reported. Further, in the present case, no formal fungal culture was done on the tissue sample and COVID-19 load was not tested continuously.

Nevertheless, physicians should be aware of the increased risk of atypical infectious diseases which may co-occur with COVID-19 infection. In patients with oncologic history, these atypical infections may mimick malignant disease progression. New abnormal lesions in a cancer patient who has had a recent COVID-19 infection should be carefully investigated with consideration of tissue biopsy in order to avoid misdiagnosis.

## Data Availability Statement

The original contributions presented in the study are included in the article/supplementary material, further inquiries can be directed to the corresponding author/s.

## Ethics Statement

Ethical review and approval was not required for the study on human participants in accordance with the local legislation and institutional requirements. The patients/participants provided their written informed consent to participate in this study. Written informed consent was obtained from the individual(s) for the publication of any potentially identifiable images or data included in this article.

## Author Contributions

EN: data curation, writing—original draft, and visualization. NM: validation, resources, and visualization. AC: conceptualization, validation, investigation, writing—review, and editing. EK: conceptualization, validation, investigation, Writing—original draft, visualization, and supervision. All authors contributed to the article and approved the submitted version.

## Conflict of Interest

The authors declare that the research was conducted in the absence of any commercial or financial relationships that could be construed as a potential conflict of interest.

## Publisher's Note

All claims expressed in this article are solely those of the authors and do not necessarily represent those of their affiliated organizations, or those of the publisher, the editors and the reviewers. Any product that may be evaluated in this article, or claim that may be made by its manufacturer, is not guaranteed or endorsed by the publisher.
